# Nitric oxide releasing nanofiber stimulates revascularization in response to ischemia via cGMP-dependent protein kinase

**DOI:** 10.1371/journal.pone.0303758

**Published:** 2024-05-20

**Authors:** Kyung Hye Lee, Min-Young Song, Sora Lee, JinSun Park, Jung Hee Kang, Haneul Cho, Ki-Bum Kim, Soo Ji Son, Xian Wu Cheng, Young Ju Lee, Gi-Ja Lee, Jae Ho Shin, Weon Kim

**Affiliations:** 1 Department of Internal Medicine, Division of Cardiovascular, Kyung Hee University Hospital, Kyung Hee University, Seoul, Korea; 2 Department of Biotechnology, Cha University, Pocheon, Korea; 3 Division of Cardiology, Asan Medical Center, University of Ulsan College of Medicine, Seoul, Korea; 4 Department of Chemistry, Kwangwoon University, Seoul, Korea; 5 Department of Cardiology and Hypertension, Jilin Provincial Key Laboratory of Stress and Cardiovascular Diseas, Yanbian University Hospital, Yanji, China; 6 Department of Biomedical Engineering, College of Medicine, Kyung Hee University, Seoul, Korea; CSIR-Indian Institute of Chemical Biology, INDIA

## Abstract

Nitric oxide (NO) promotes angiogenesis via various mechanisms; however, the effective transmission of NO in ischemic diseases is unclear. Herein, we tested whether NO-releasing nanofibers modulate therapeutic angiogenesis in an animal hindlimb ischemia model. Male wild-type C57BL/6 mice with surgically-induced hindlimb ischemia were treated with NO-releasing 3-methylaminopropyltrimethoxysilane (MAP3)–derived or control (i.e., non-NO-releasing) nanofibers, by applying them to the wound for 20 min, three times every two days. The amount of NO from the nanofiber into tissues was assessed by NO fluorometric assay. The activity of cGMP-dependent protein kinase (PKG) was determined by western blot analysis. Perfusion ratios were measured 2, 4, and 14 days after inducing ischemia using laser doppler imaging. On day 4, Immunohistochemistry (IHC) with F4/80 and gelatin zymography were performed. IHC with CD31 was performed on day 14. To determine the angiogenic potential of NO-releasing nanofibers, aorta-ring explants were treated with MAP3 or control fiber for 20 min, and the sprout lengths were examined after 6 days. As per either LDPI (Laser doppler perfusion image) ratio or CD31 capillary density measurement, angiogenesis in the ischemic hindlimb was improved in the MAP3 nanofiber group; further, the total nitrate/nitrite concentration in the adduct muscle increased. The number of macrophage infiltrations and matrix metalloproteinase-9 (MMP-9) activity decreased. Vasodilator-stimulated phosphoprotein (VASP), one of the major substrates for PKG, increased phosphorylation in the MAP3 group. MAP3 nanofiber or NO donor SNAP (s-nitroso-n-acetyl penicillamine)-treated aortic explants showed enhanced sprouting in an *ex vivo* aortic ring assay, which was partially abrogated by KT5823, a potent inhibitor of PKG. These findings suggest that the novel NO-releasing nanofiber, MAP3 activates PKG and promotes therapeutic angiogenesis in response to hindlimb ischemia.

## Introduction

Peripheral artery disease (PAD) resulting from atherosclerosis is on the rise in the elderly, with a prevalence of more than 20% [1]. Thus the global burden of the disease is increasing, and this trend is expected to be maintained [2]. Some methods, such as endovascular procedures or surgical revascularization, are the first choice for treating lifestyle-limiting claudication or hemodynamically significant stenosis [3]; however, even endovascular therapy is often too invasive for frail elderly patients. There are some patients who have a diabetic coronary artery disease and severe PAD, making it difficult to treat invasive treatment.

Nitric oxide (NO) is a crucial signaling molecule that modulates various physiological and pathological processes, including platelet aggregation, inflammation, angiogenesis, and recovery from ischemic injuries [4–8]. The role of NO in angiogenesis has been studied for a long time and has shown that exogenous administration of a novel NO donor stimulates the proliferation of cultured rat aortic endothelial cells [9]. Furthermore, defective endothelial NO synthesis may limit angiogenesis in patients with atherosclerosis-related endothelial dysfunction [10].

Despite the pro-angiogenetic effect of NO, the high reactivity of NO necessitates the development of an NO delivery system. However, an effective NO delivery system has not yet been established for ischemic disease. NO-releasing nanofibers have the advantage of controlling the amount and time of NO release. In addition, it allows NO to easily penetrate the skin and tissues; thus, NO can act directly at the target lesion, thereby minimizing side effects.

In the present study, we investigated the angiogenic effect of locally administered NO-releasing fibers under ischemic conditions and examined the potential involvement of protein kinase (PKG) signals in the pathway of NO-regulated neo-vascularization.

## Materials and methods

### Animals and experimental protocol

Male wild-type mice with a C57BL/6 background aged 8–10 weeks were used as the model in this study. The study protocol was approved by the Institutional Animal Care and Use Committee (IACUC) of the Kyung Hee University Hospital (approval number: KHMC-IACUC 2017–013). We used a mouse model of neo-vascularization, in which, as per standard, the entire left femoral artery and vein were surgically removed under anesthesia with ketamine and Rompun (i.p.). The mice were randomly divided into three groups and treated with NO-releasing 3-methylaminopropyltrimethoxysilane–derived nanofiber (MAP3), Non-NO-releasing nanofiber (Control nanofiber) or left untreated (Ischemia). MAP3 or control nanofibers were applied for 20 min onto the surface of the wound three times every two days after the operation (S1 Fig). All the ischemic hindlimbs were shaved and applied with the same size of nanofiber sheets.

### Preparation of NO-releasing MAP3-derived nanofibers

The synthetic scheme of NO-releasing MAP3-derived nanofibers was described in S2 Fig. An aminoalkoxysilane solution was prepared by dissolution of 5.0 mmol of MAP3 in 3.0 ml of ethanol in the presence of sodium methoxide (5.0 mmol; an equimolar amount corresponding to the secondary amine content of the silane precursor) [11,12]. The solution was placed in an in-house NO reactor and flushed with Ar for 10 min, followed by a series of three charge/discharge cycles with Ar (10 atm, 3 x 10 min) to remove oxygen in the solution. The reaction bottle was then charged with NO (99.99%) to 10 atm and sealed for 3 days at room temperature while being stirred. Prior to removal of the *N*-diazeniumdiolate (NO donor moiety)-modified aminoalkoxysilane (Component I) solution, unreacted NO was purged from the chamber with Ar gas for 10 min. *N*-Diazeniumdiolate-modified MAP3 solution was vacuum sealed and stored in –20°C freezer until use.

A mixture of 30.0 mmol of methylmethacrylate (MMA), 10.0 mmol of hexylmethacrylate (HMA), and 10.0 mmol of 3-(trimethoxysilyl)propyl methacrylate (SiMA) were added to 30.0 ml of toluene with stirring, and then the mixed solution was heated until 80°C. Polymerization reaction of methacrylate mixture was initiated by 0.1 mmol of azobisisobutyronitrile (AIBN) (dissolved in 2.5 ml of methanol) and reacted for 12 h. After the reaction was completed, toluene was vaporized by vacuum distillation at 60°C and vacuum drying at room temperature. Residual monomers and AIBN were washed copiously with hexane 3 times and the product was vacuum dried again. The synthesized poly(MMA-co-HMA-co-SiMA) (Component II) was stored with vacuum packaging until use.

Poly(MMA-co-HMA-co-SiMA) (0.3 g, Component II) was dissolved in 0.7 g of acetone/ dimethylformamide (DMF) (3:1 in volume). 143 μL (1.0 mmol) of methyltrimethoxysilane (MTMOS, Component III) and 250 μL (0.25 mmol) of *N*-diazeniumdiolate-modified MAP3 (Component I) were added to 1.0 g of poly(MMA-co-HMA-co-SiMA) solution. 19.0 mg of aluminum acetylacetonate dissolved in 400 μL of acetone/DMF (3:1 in volume) and 30 μL H_2_O was consecutively added to the solution of poly(MMA-co-HMA-co-SiMA), MTMOS, and *N*-diazeniumdiolate-modified MAP3. The mixture was then stirred for 1 h at 4°C to minimize any thermal decomposition of *N*-diazeniumdiolate NO donors. After stirring, the mixed solution was electrospun immediately.

Nitric oxide storage/delivery nanofibers were prepared by the electrospinning system (ESR200RD, NanoNC; Seoul, Korea) and Harvard PHD 2000 syringe pump (Holliston, MA, USA). The mixed solution prepared above was loaded in a plastic syringe and flowed through a 25-G needle. The electrospinning conditions were as follows: 10 μL/min of the volume flow rate, 23.5 kV of the applied voltage, 15 cm of tip to collector distance, and 30 min of the electrospinning time. After stabilization in air for 30 min, NO storage/delivery nanofiber webs were vacuum sealed and stored in –20°C freezer.

### Characterization of NO-releasing nanofibers

The surface morphologies of the electrospun nanofibers were characterized by using a scanning electron microscope (SEM). Nitric oxide storage/delivery nanofiber webs were sputter-coated with Pt and imaged using a Hitachi S4700 (Tokyo, Japan). Fiber diameters were averaged from at least 15 measurements.

Nitric oxide release profiles of the electrospun nanofibers was monitored in deoxygenated phosphate-buffered saline (PBS; 0.01 M, pH 7.4) at 37°C using a Sievers NOA 280i Chemiluminescence nitric oxide analyzer (Boulder, CO, USA) [11,12]. Nitric oxide released from the nanofiber was transported to the analyzer by a stream of Ar gas (70 ml·min^-1^) passed through the reaction cell. The instrument was calibrated with air passed through a zero filter (0 ppm NO) and 45 ppm of NO standard gas (balance N_2_). Nitric oxide release kinetics of MAP3 nanofiber was determined in terms of total amount of NO release, t[NO]; maximum flux of NO release, [NO]_m_; time necessary to reach [NO]_m_, *t*_m_; half-life of NO release, *t*_1/2_; and, duration time of NO release for sustained fluxes of NO ≥ 1 ppb/mg, *t*_d_.

#### Cell culture and cell viability measurements

Mouse L929 cells were obtained from the Korea Cell Line Bank (Seoul, South Korea). The cells were routinely maintained in complete growth medium consisting of RPMI 1640 (GIBCO, Grand Island, NY, USA) supplemented with L-glutamine, 20 mM 4-(2-hydroxyethyl)piperazine-1-ethane-sulfonic acid (HEPES), 25 mM NaHCO_3_, 10% heat-inactivated fetal bovine serum (FBS), and 1% penicillin/streptomycin at 37°C in an incubator with a humidified 5% CO_2_ atmosphere. Cells (2 × 10^5^ cells per ml) were seeded in a 24-well plate and incubated for 24 h to examine cell cytotoxicity. Cells were directly treated with various weights (0, 0.5, 1, and 2 mg) of control (without NO) or NO-releasing nanofibers, and were then further incubated at 37°C in a 5% CO_2_ incubator for 24 h. Cell toxicity was measured using an EZ-CyTox colorimetric cell viability assay kit (water-soluble tetrazolium salt [WST] assay, DaeilLab, Korea). The optical density at 450 nm was recorded by a multi-microplate reader (Synergy HT multi-mode microplate instrument, BioTek, Winooski, VT, USA).

### Laser doppler image analysis

The ratio of ischemic hindlimb blood flow was determined using laser doppler blood flowmetry (LDBF; MoorLDI, Moor Instruments, Devon, UK). LDBF analyses were performed on the legs and feet of the mice before and after surgery. Laser doppler perfusion image (LDPI) was performed on days 0, 2, 4, and 14 following femoral artery ligations. The LDPI ratio was determined as the ratio of ischemic to normal hindlimb blood flow.

### Capillary density analysis

Capillary density in the adductor muscle was analyzed to obtain specific evidence of vascularity at the microcirculation level. Tissue samples were obtained from the ischemic thigh adductor skeletal muscles on postoperative day 14. Frozen tissue sections of 5 μm thickness were prepared from each sample. Capillary endothelial cells were identified by immunohistochemical staining with a CD31 polyclonal antibody (Ab28364; Abcam, Cambridge, MA, USA). TRITC-conjugated secondary antibody for detecting CD31, WGA-FITC conjugates and DAPI applied. Fifteen random microscopic fields from three different sections in each tissue block were examined for the presence of capillary endothelial cells, expressed as the ratio of the number of capillaries to the number of myofibers per high-power field (×400).

### Macrophages infiltration analysis

To determine whether the increase in capillary density was due to inhibition of inflammation, tissue macrophages were analyzed on postoperative day 4. Paraffin tissue sections of 5-μm thickness were prepared from each sample and stained with anti-F4/80 monoclonal antibody (Abcam 228155, USA). Sections were visualized with DAB and eosin counter stain. Fifteen random microscopic fields from three different sections in each tissue block were examined, and the infiltrated macrophages were expressed as the ratio of F4/80^+^ cell number to myofibers per high power field (magnification, × 400).

### Zymography and western blot analysis

Tissue samples obtained on post-operative day 4 were homogenized with TissueLyser II (Qiagen, Hilden, Germany). in PRO-PREP^™^ Protein extraction solution (iNtRON Biotechnology, Inc. Korea) containing PMSF, EDTA, pepstatinA, leupeptin, aprotinin. Protein content was determined by the BCA (Sigma Chemical Co, St, Louis, MO, USA), and the same amounts of non-reduced protein sample were separated with gelatin-contained SDS- polyacrylamide gels. After the removal of SDS from the gels, gelatinase activity was developed in the buffer containing 1% Triton X-100, 50 mM Tris-HCl, 5 mM CaCl_2_, 1 μM ZnCl_2_ for 24 hours at 37°C with agitation. The gels were stained with Coomassie blue, and the matrix metalloproteinase (MMP)-9 and MMP-2 activity was measured from gelatinolytic clear bands. Protein homogenates obtained from post-operative 20 min were separated by SDS-PAGE and the transferred to a PVDF (polyvinylidene fluoride, Millipore, MA, USA) membrane. The membranes were immune-blotted with the anti-vasodilator-stimulated phosphoprotein (VASP) or anti- phosphor-VASP (Ser239) polyclonal antibodies (Cell signaling technology, MA, USA) at a 1:1000 dilution, followed by secondary antibody (Cell signaling technology) at a 1:5000 dilution. Bands were visualized, using the chemiluminescence SuperSignal^™^ West Femto (Thermo Scientific, Rockford, IL, USA).

### Aorta sprouting assay

Aortas were harvested from C57BL/6 mice and sectioned into 1-mm long segments. The rings were embedded in a 48-well culture dish with 100 μl Matrigel (BD Biosciences, CA, USA) and maintained in EGM2 medium (Lonza, Walkersville, USA). The cultures were treated with KT5823 (1 μM, TOCRIS, UK) prior to applying MAP3 or *S*-nitroso-*N*-acetyl penicillamine (SNAP) (1 mM, Sigma, USA) in 2% FBS contained EGM-2. MAP3 nanofiber applied with same size (70 mm × 70 mm) for 20 min and then removed. After 6 days, the vascular branch points were counted.

### Total NO assay

Fresh tissues obtained from post-operative 20 min were homogenized with ice-cold PBS immediately after sacrifice. Ten microliters of ultrafiltered-homogenates through a 30 kDa molecular weight cut-off filter was used for the determine the nitrate or nitrite concentration using Nitrate / Nitrite Fluorometric assay kit (Cayman Chemical, MI, USA). Total NO amounts were calculated as pmol of total nitrate per μg of tissue weight.

### Statistical analysis

Data are presented as the mean ± standard error of the mean. All data were subjected to one-way analysis of variance (ANOVA), followed by Newman-Keuls multiple comparison test. P values < 0.05 were considered to be statistically significant.

## Results

### Properties of NO-release nanofiber

**Fig 1:**
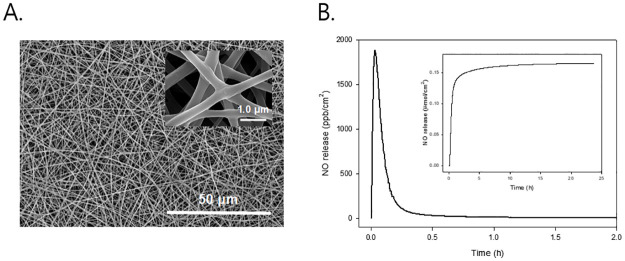
Properties of NO-release nanofibers. (A) shows SEM images of NO-releasing electospun MAP3 nanofiber. Inset represents an enlarged (10-fold) view. (B) shows real-time NO release profile for 2 h in phosphate buffered saline (0.01 M, pH 7.4) at 37°C. Inset represents total NO release amount, t[NO]. NO: Nitric oxide; SEM: Scanning electron microscope; MAP3: 3-methylaminopropyltrimethoxysilane.

Fig 1A. shows the SEM image of NO-releasing MAP3 nanofibers. The surface morphology of electrospun nanofiber exhibited a well-developed fibrous shape and clean surface with average diameters of 370 ± 50 nm. The NO storage and release properties of the electrospun nanofibers were evaluated under physiological conditions (PBS; pH 7.4 at 37 °C). A real-time NO release profile and total NO amount (inset) released from MAP3 nanofiber per unit area (1.0 cm^2^). The NO release from the *N*-diazeniumdiolate-modified MAP3-based nanofibers was characterized by a relatively fast and short release (i.e., *t*_1/2_ = 5.0 ± 0.6 min and *t*_d_ = 24 ± 1.2 h) with a high concentration of NO (i.e., t[NO] = 0.17 ± 0.025 μmol/cm^2^ and [NO]_m_ = 1870 ± 200 ppb/cm^2^) (Fig 1B).

### Cytotoxicity of nanofibers

To evaluate the biocompatibility of NO-releasing nanofibers, we performed cytotoxicity assays on control and NO-releasing nanofibers using mouse L929 cells (S3 Fig). L929 cells were treated with various weights (0 to 2 mg) of control and NO-releasing MAP3 nanofibers, and cell viability was analyzed by WST assay. As a result, both control and NO-releasing nanofibers did not show severe cytotoxic effects on L929 cells up to a weight of 1 mg. Importantly, the amount of nanofibers electrospun on per sheet (10 mm × 20 mm) was 0.9 mg.

### NO fiber promotes perfusion in hindlimb ischemia and increases capillary density

**Fig 2:**
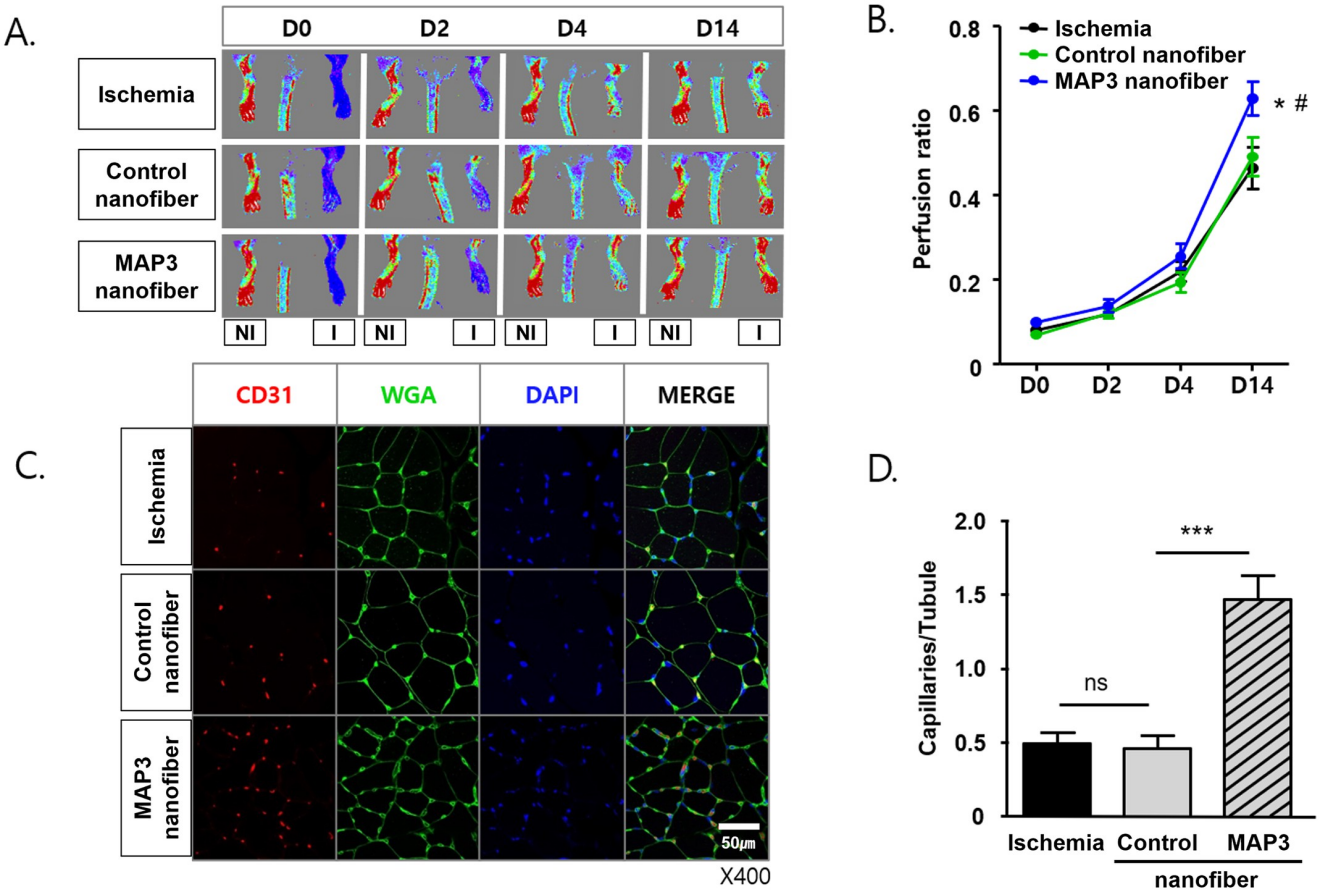
MAP3 improves perfusion recovery and increases capillary density in hindlimb ischemia. (A) Representative laser Doppler perfusion images (LDPI) for the untreated (Ischemia), Non-NO-releasing nanofiber (Control nanofiber) and NO-releasing nanofiber (MAP3 nanofiber) applied mice plantar. These images were obtained on day post-ischemia 0, 2, 4 and 14. (B) The perfusion ratio of ischemic (left) /non-ischemic (right) limb was measured from each group, Ischemia (n = 11), Control (n = 9), MAP3 (n = 11). (C) Representative immunofluorescent images stained with CD31-TRITC, WGA-FITC, DAPI from the adduct muscle cryo-sections on day 14 post-ischemia. (D) The number of CD31^+^DAPI^+^ capillaries on WGA green fluorescence were counted and presented as numbers of capillaries per WGA-surrounded myotubules. Error Bars indicate mean ± standard error of the mean. *p<0.05, ***p<0.001. MAP3: 3-methylaminopropyltrimethoxysilane; WGA: Wheat germ agglutinin; FITC: Fluorescein isothiocyanate; I: Ischemic; NI: Non-ischemic.

The blood flow to the ischemic hindlimb of the MAP3 nanofiber group measured by LDBF increased gradually, and the differences between groups were definite on day 14 (Fig 2A). The LDPI ratio was higher in the MAP3 nanofiber group than in the control nanofiber and ischemia groups (*p*<0.05) (Fig 2B). Immunohistochemical staining with CD31 show the number of capillaries was higher in the MAP3 nanofiber group than in the control nanofiber and ischemia groups (*p*<0.001). There was no significant difference in the ratio between the control nanofiber and ischemia groups (*p<0.05, ***p<0.001) (Fig 2C and 2D).

### NO fiber regulates macrophages infiltration and inactivates MMP-9

**Fig 3:**
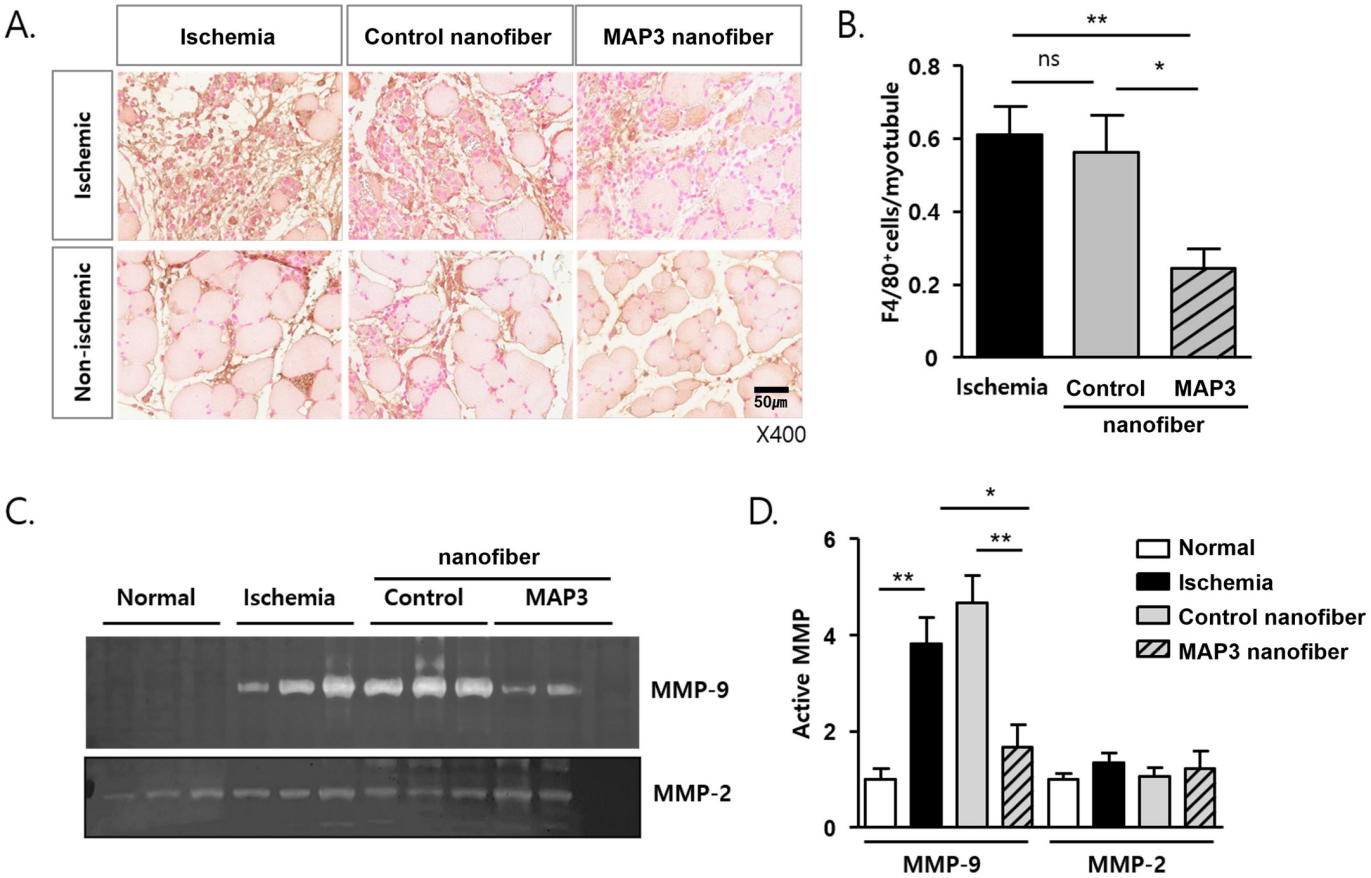
MAP3 ameliorates macrophage infiltration and MMP9 activity. (A) Representative images of immunohistochemical stain with F4/80 and eosin from paraffin-sections of day 4 post-ischemia. Both of ischemic wound area and non-ischemic area were observed. (B) F4/80 positive macrophages of sections from non-ischemic area were counted and presented as numbers of F4/80^+^/myotubules. (C) Gelatin-zymography were performed with same amount of proteins from tissue homogenates of day 4 post-ischemia. Representative MMP-9 and MMP-2 gelatinolytic images were shown. Age matched normal mice tissues were used (Normal). (D) MMP-9 and MMP-2 activities were analyzed with the clear band and the relative activities to normal were shown as mean ± standard error of the mean. *p<0.05, **p<0.01. MAP3: 3-methylaminopropyltrimethoxysilane; MMP: Matrix metalloproteinase.

The infiltration of F4/80+ macrophages was decreased by NO-releasing nanofibers 4 days after ischemic insult, and the number of F4/80+ macrophages per myofiber was reduced by the NO nanofiber, as determined by counting the number of positive cells in the non-ischemic area (*p<0.05, **p<0.01) (Fig 3A and 3B). MMP-9 activity decreased in the MAP3 nanofiber group than in the control nanofiber and ischemia groups (p<0.01, p<0.05) (Fig 3C and 3D). However, the activity of MMP-2 was similar in all groups (Fig 3C and 3D).

### NO fiber induces aorta sprouting via PKG and releases NO into damaged tissues

**Fig 4:**
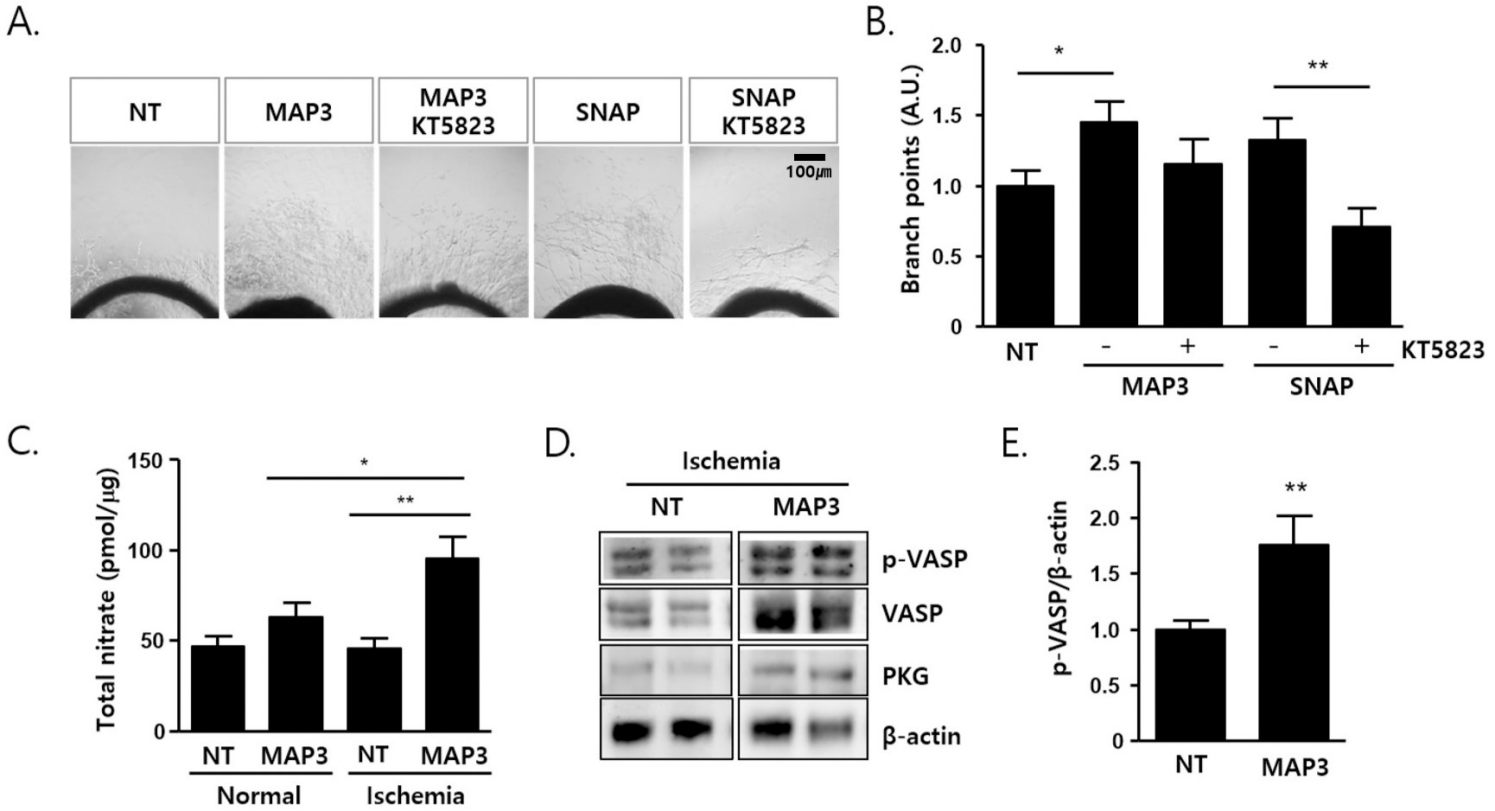
MAP3 induces aorta sprouting via PKG and releases NO into damaged tissues. (A) Matrigel embedded rat aorta ring were pretreated with KT5823 (1 μM, 30 min) and followed by same size of MAP3 (70 mm × 70 mm) for 20 min or SNAP (1 mM). The images were captured after 6 days culture. (B) Branch points of aorta sprout were counted from 3 independent experiment and shown as number of branch point relative to NT. (C) MAP3 were applied on left limb of normal or hindlimb ischemic mice (MAP3), while right limb of normal mice (NT/Normal) and left limb of ischemic mice (NT/Ischemia) were applied nothing but shaving. Release NO amount of MAP3 into muscle tissue were determined from adduct muscles and shown as total nitrate (pmol) per tissue homogenates (μg). (D) Tissue homogenates as shown in (C) were immunoblotted with anti-p-VASP Ab. (E) The PKG activity was quantified with VASP phosphorylation in MAP3 and compared with no-treated ischemic hindlimb tissues. Error Bars indicate mean ± standard error of the mean. *p<0.05, **p<0.01. NT: No-treat group; MAP3: 3-methylaminopropyltrimethoxysilane; SNAP: S-nitroso-n-acetyl penicillamine; MAP3: 3-methylaminopropyltrimethoxysilane; VASP: Vasodilator-stimulated phosphoprotein; PKG: Protein kinase.

NO induces various cellular responses by increasing cyclic GMP levels and consequently activating PKG. An aortic ring culture assay was performed to confirm NO ex vivo direct angiogenesis. Applying NO-releasing nanofibers or SNAP-treated aortic explants showed enhanced sprouting in an ex vivo aortic ring assay, which was partially abrogated by KT5823, a potent inhibitor of PKG. We found that aorta sprout remnants decreased after treatment with the PKG inhibitor KT5823 (Fig 4A and 4B). The total nitrate/nitrite concentration in the adduct muscle increased 20 min after application of the nanofiber onto the skin in the muscle of ischemic limb compared with control group (p<0.01). Interestingly, NO concentration did not increase non-ischemia limb compared with ischemic-limb in the MAP 3 group (p<0.05) (Fig 4C). VASP phosphorylation at Ser 239, a substrate of PKG, was elevated in the muscle subjected to NO-releasing fibers (**p<0.01) (Fig 4D and 4E).

## Discussion

This study was designed to evaluate NO-releasing nanofibers as a treatment option for PAD, using an ischemic hind limb model and 3% MAP3 nanofibers, attachable to the skin. The present study showed that 3% MAP3 NO-releasing nanofiber effectively increased local NO concentration in the target lesion and induced angiogenesis. Treatment with 3% MAP3 resulted in angiogenesis, as proven by the increased limb perfusion and capillary density compared with those observed in control nanofiber or untreated mice. These results correspond well with those of previous experimental studies [13]. Sufficiently elevated NO concentration in the target tissue reduced inflammation by inhibiting macrophage infiltration and decreasing MMP-9 activity. These findings are inconsistent with the results of previous studies that show that NO and reactive nitrogen species can induce inflammation by activating MMP-9 [14,15]. The results of the present study were consistent with those of earlier studies that proposed that administration of high NO concentrations can inhibit MMP-9 activity and expression [14,16,17]. This disparity is believed to be due to the different NO concentrations. Furthermore, the *in vivo* and *in vitro* results of this experiment demonstrated that angiogenesis generated by the NO-releasing nanofibers was mediated by PKG activation that increased angiogenesis through the phosphorylation of serine 239 of VASP.

### Effects of NO in peripheral arterial disease

NO is a chemical messenger that regulates numerous intracellular signaling pathways, including vasodilation, wound healing, and angiogenesis. NO is a gaseous molecule with an ultra-short half-life, and it is therefore difficult to maintain an appropriate level for treatment. Although many preclinical studies have indicated that NO can induce angiogenesis and vasodilation under ischemic conditions [13,18–20], most studies have not proven the efficacy of NO for PAD in human clinical trials [21–23]; and thus far, no treatments utilizing NO have been approved for clinical use in patients with PAD [24].

There are some important prerequisites for ensuring clinical application of NO and designing its delivery system in humans. Firstly, since NO is a gaseous molecule with high reactivity and an ultra-short half-life, the NO-delivery system must be capable of stabilizing this reactivity and release NO slowly to maintain an effective therapeutic concentration. A previous study described that NO donors with carrier nanoparticles were more effective than NO donors alone, suggesting that sustained NO release rather than excessive NO concentration was crucial to restore under ischemia and prevent reactive oxygen species (ROS) stress, including peroxynitrite (ONOO-) [18]. Secondly, to avoid nanoparticles persisting in the body resulting in a higher than acceptable systemic concentration, a less invasive route of administration route is needed. Finally, nitrate tolerance should be considered for continuous use in chronic diseases such as PAD.

### NO release systems

MAP3 is an organic exogenous NO delivery system. Among the other NO donor nanoparticles, MAP3 has been studied for a long time, and it’s NO release profile is adjusted by mol% [25,26]. This nanoparticle has also been demonstrated as a non-toxic carrier with large NO payloads in previous *in vitro* experimental studies [19,26]. In the current study, the drug was administrated by cutaneous contact, thus preventing systemic side effects and maximizing local concentration. The NO concentration after applying 3% MAP3 peaked within 10 min and decreased within 20 min. The peak concentration of approximately 3200 ppb/mg was insufficient to aggravate ROS stress while appearing to be suitable to enhance the anti-inflammatory effect and angiogenesis. The experiments of Schirmer et al [27] were similar to this one in terms of study design to prove the anti-ischemic effect of the NO-donor, MPC-1011, with the major difference being that the administration route was an implantable subcutaneous infusion pump. Nevertheless, the administration methods were too invasive for patients. Gene therapy remains a future possibility for treatment of this disease. There are still many questions to be answered, such as the mechanism of NO signaling dysregulation in vascular disease, interactions between superoxide and NO, and mechanism of long-term gene transfer vectors devoid of toxic inflammatory effects [28].

There are some limitations to our study. First, the mice hindlimb ischemic model resulting from surgical excision of the entire left femoral artery and vein is insufficient to reflect the pathophysiology and clinical features of critical limb ischemia. Second, it was not a clinical trial in humans, and the results cannot be extrapolated as it is. In addition, it is difficult to modulate various risk factors of atherosclerosis, including hypertension, smoking, diabetes, and dyslipidemia in animal models. Third, the NO releasing capacity of MAP3 can be regulated by mol % according to desired therapeutic activity, such as anti-tumor, anti-bacterial, and anti-inflammatory effects. Before conducting a trial in humans, it is necessary to establish the treatment efficacy and side effects of PAD at various concentrations. We are currently planning to conduct a trial in patients after collecting more data on various concentrations of NO donors.

## Conclusions

NO-releasing nanofibers play a noteworthy role in the functional recovery from ischemia and activation of neovascularization and would therefore be applied as a new treatment option for patients with PAD.

## Supporting information

S1 Fig Study diagram. Control or MAP3 nanofibers were applied to the wound surface 20 minutes after surgery three times for every two days, and LDPI observed on days 0, 2, 4, and 14 following femoral artery ligations. MAP3: 3-methylaminopropyltrimethoxysilane; LDPI: Laser doppler perfusion image.
(TIF)

S2 Fig Synthetic scheme of NO-releasing MAP3-derived nanofibers. To address leaching concerns of NO donors and their carcinogenic decomposition byproducts (e.g., diamines and corresponding nitrosamines) into the biological media, our efforts were devoted to covalently tether the NO donor agents to the polymer backbone. The trimethoxysilyl moiety of each component (i.e., Components I, II, and III) contributes to the formation of sol-gel networks during the electrospinning process. Eventually, the NO donor *N*-diazeniumdiolate moieties are covalently attached to the backbone of the nanofibers.
(TIF)

S3 Fig Cytotoxicity measurements of nanofibers. To evaluate the biocompatibility of MAP3-derived nanofibers, cytotoxicity assays were performed on control and NO-releasing MAP3 nanofibers using mouse L929 cells. L929 cells (2 × 10^5^ cells per ml) were directly treated with various weights (0, 0.5, 1, and 2 mg) of control (without NO) and NO-releasing nanofibers. Cell toxicity is expressed as a percentage of the normal control without control fiber or NO-releasing fiber.
(TIF)

S1 Data (ZIP)
