# Correction: Nitric oxide releasing nanofiber stimulates revascularization in response to ischemia via cGMP-dependent protein kinase

**DOI:** 10.1371/journal.pone.0314132

**Published:** 2024-11-14

**Authors:** Kyung Hye Lee, Min-Young Song, Sora Lee, JinSun Park, Jung Hee Kang, Haneul Cho, Ki-Bum Kim, Soo Ji Son, Xian Wu Cheng, Young Ju Lee, Gi-Ja Lee, Jae Ho Shin, Weon Kim

The funding statement for this paper is incorrect. The correct funding statement is as follows: This research was supported by a grant from the National Research Foundation (grant number 2022R1A2C2008867), awarded to Prof. Weon Kim.
