# Supplementary figures and images for: Nitric oxide releasing nanofiber stimulates revascularization in response to ischemia via cGMP-dependent protein kinase
Source: PLoS One. 2024 May 20;19(5):e0303758. doi: 10.1371/journal.pone.0303758 (PMC11104631; doi:10.1371/journal.pone.0303758)

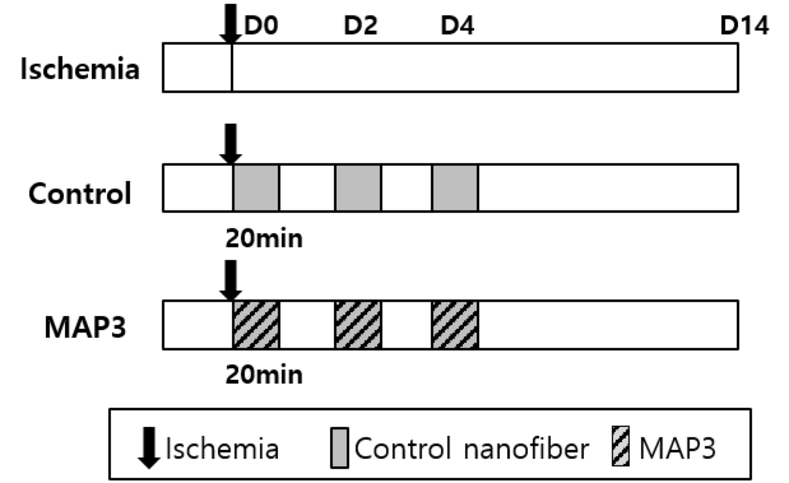

Supplement: S1 Fig — Control or MAP3 nanofibers were applied to the wound surface 20 minutes after surgery three times for every two days, and LDPI observed on days 0, 2, 4, and 14 following femoral artery ligations. MAP3: 3-methylaminopropyltrimethoxysilane; LDPI: Laser doppler perfusion image. (TIF) [file pone.0303758.s001.tif]

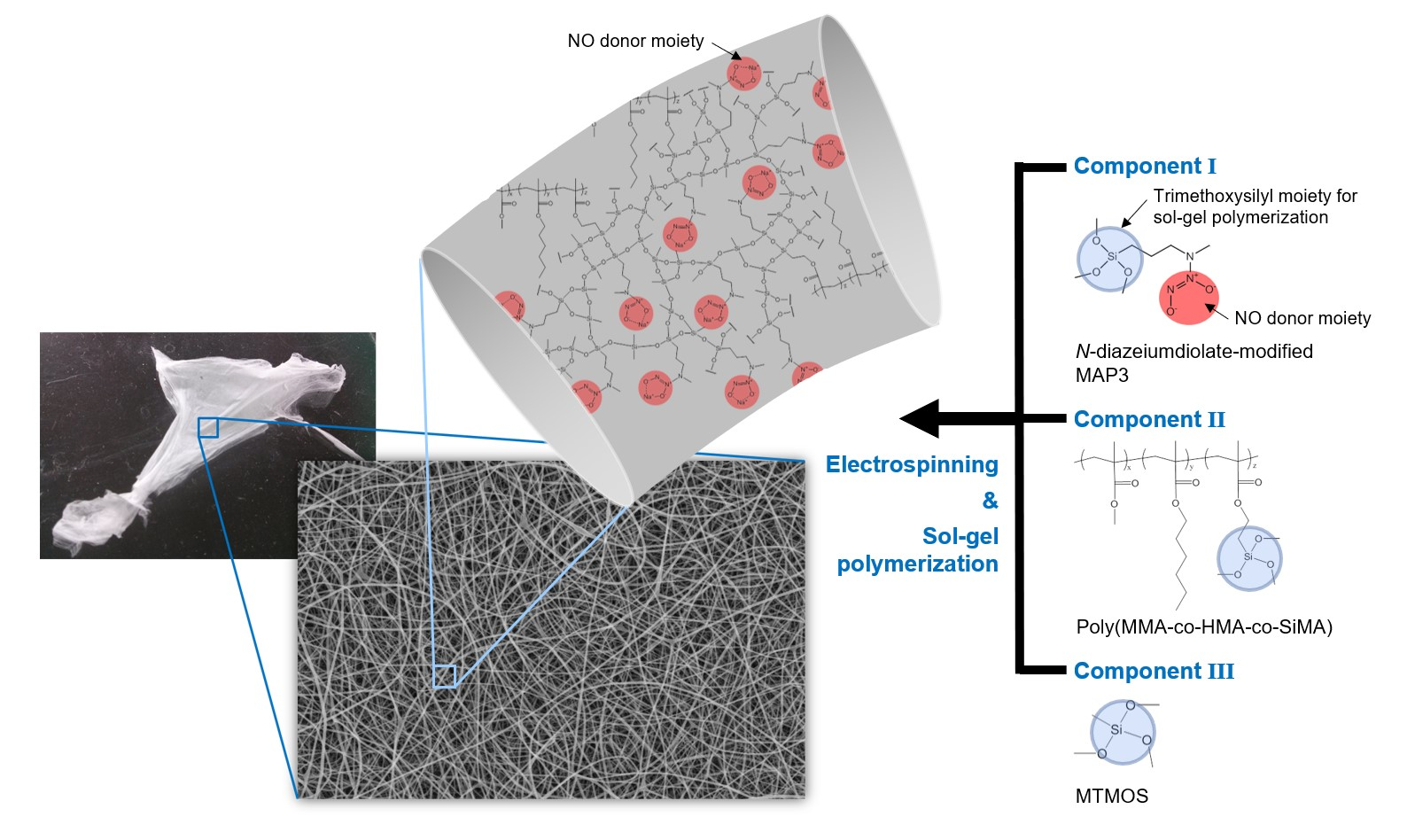

Supplement: S2 Fig — To address leaching concerns of NO donors and their carcinogenic decomposition byproducts (e.g., diamines and corresponding nitrosamines) into the biological media, our efforts were devoted to covalently tether the NO donor agents to the polymer backbone. The trimethoxysilyl moiety of each component (i.e., Components I, II, and III) contributes to the formation of sol-gel networks during the electrospinning process. Eventually, the NO donor N-diazeniumdiolate moieties are covalently attached to the backbone of the nanofibers. (TIF) [file pone.0303758.s002.tif]

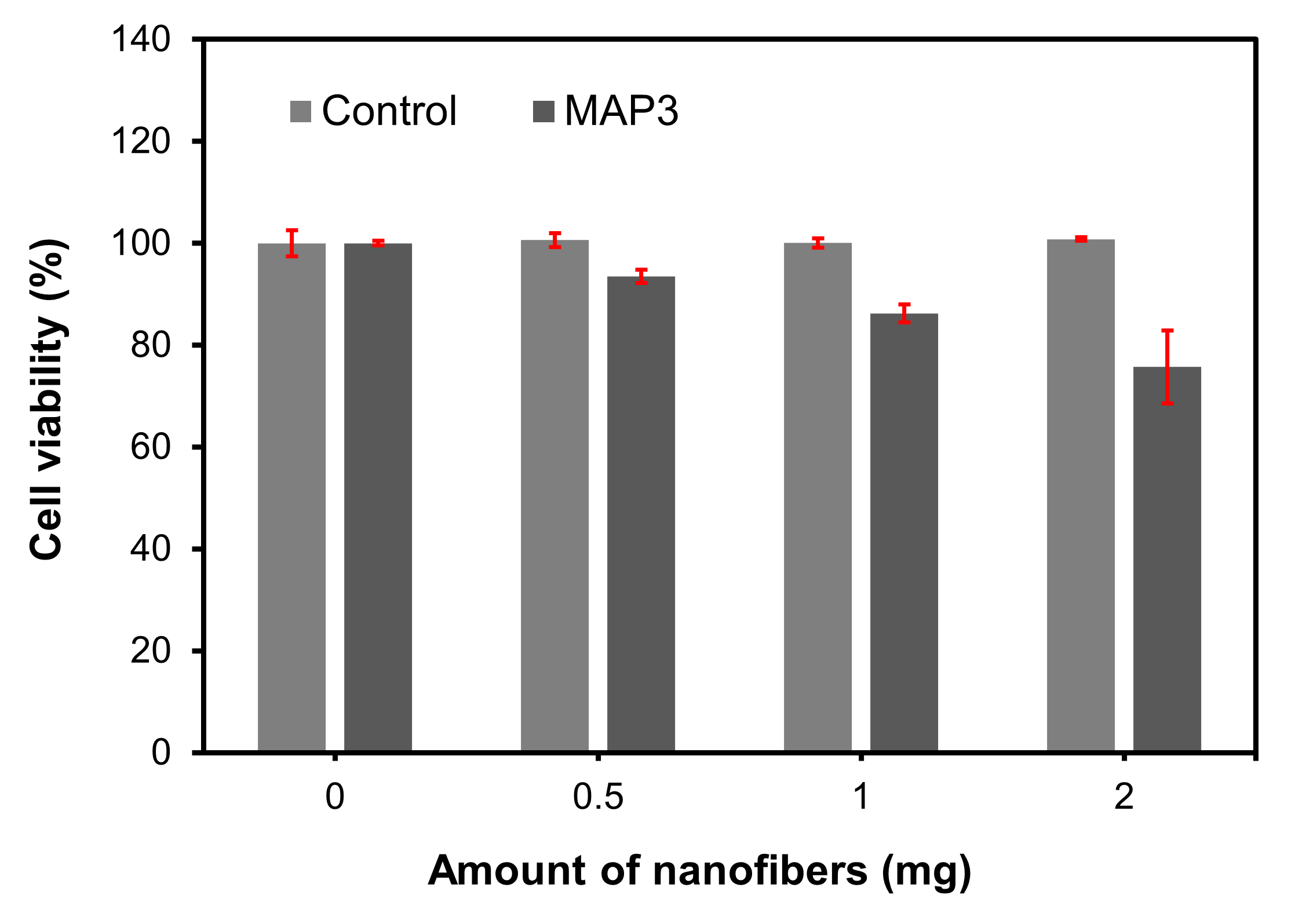

Supplement: S3 Fig — To evaluate the biocompatibility of MAP3-derived nanofibers, cytotoxicity assays were performed on control and NO-releasing MAP3 nanofibers using mouse L929 cells. L929 cells (2 × 10^5^ cells per ml) were directly treated with various weights (0, 0.5, 1, and 2 mg) of control (without NO) and NO-releasing nanofibers. Cell toxicity is expressed as a percentage of the normal control without control fiber or NO-releasing fiber. (TIF) [file pone.0303758.s003.tif]

Figure 3C. Uncropped blots

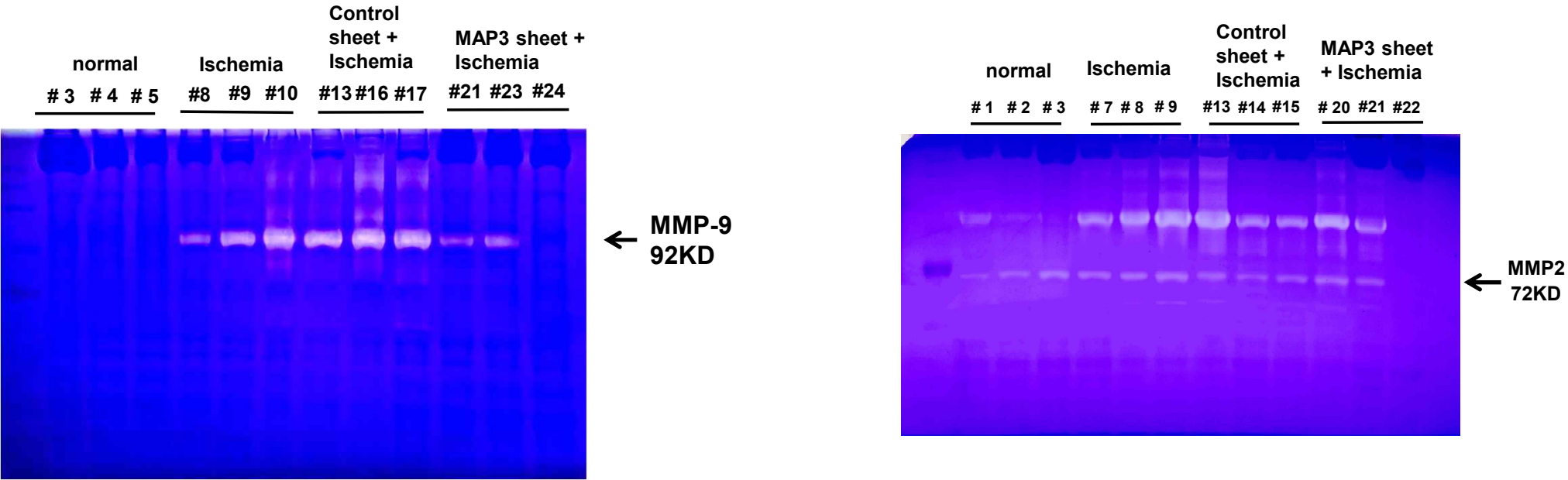

Supplement: S1 Data — (ZIP) [file pone.0303758.s004.zip › Figure 3C_MMP9_MMP2_gel image_raw.pdf]
